# Locomotor Exercise Enhances Supraspinal Control of Lower-Urinary-Tract Activity to Improve Micturition Function after Contusive Spinal-Cord Injury

**DOI:** 10.3390/cells11091398

**Published:** 2022-04-20

**Authors:** Lingxiao Deng, Tao Sui, Dong V. Wang, Shaoping Hou, Xiaojian Cao, Kaiwen Peng, Zaocheng Xu, Xiaoming Xu

**Affiliations:** 1 Spinal Cord and Brain Injury Research Group, Stark Neurosciences Research Institute, Indiana University School of Medicine, Indianapolis, IN 46202, USA; nydsuitao@163.com (T.S.); pkevin33@126.com (K.P.); zcxu@iupui.edu (Z.X.); 2 Department of Neurological Surgery, Indiana University School of Medicine, Indianapolis, IN 46202, USA; 3 Department of Orthopedics, The First Affiliated Hospital of Nanjing Medical University, Nanjing 210000, China; xiaojiancao001@163.com; 4 Department of Neurobiology & Anatomy, Drexel University College of Medicine, Philadelphia, PA 19129, USA; dw657@drexel.edu (D.V.W.); sh698@drexel.edu (S.H.); 5 Department of Anatomy and Cell Biology, Indiana University School of Medicine, Indianapolis, IN 46202, USA

**Keywords:** locomotor exercise, lower urinary tract, supraspinal, external urethral sphincter, contusive spinal-cord injury

## Abstract

The recovery of lower-urinary-tract activity is a top priority for patients with spinal-cord injury. Historically, locomotor training improved micturition function in both patients with spinal cord injury and animal models. We explore whether training augments such as the supraspinal control of the external urethral sphincter results in enhanced coordination in detrusor-sphincter activity. We implemented a clinically relevant contusive spinal-cord injury at the 12th thoracic level in rats and administered forced wheel running exercise for 11 weeks. Awake rats then underwent bladder cystometrogram and sphincter electromyography recordings to examine the micturition reflex. Subsequently, pseudorabies-virus-encoding red fluorescent protein was injected into the sphincter to trans-synaptically trace the supraspinal innervation of Onuf’s motoneurons. Training in the injury group reduced the occurrence of bladder nonvoiding contractions, decreased the voiding threshold and peak intravesical pressure, and shortened the latency of sphincter bursting during voiding, leading to enhanced voiding efficiency. Histological analysis demonstrated that the training increased the extent of spared spinal-cord tissue around the epicenter of lesions. Compared to the group of injury without exercise, training elicited denser 5-hydroxytryptamine-positive axon terminals in the vicinity of Onuf’s motoneurons in the cord; more pseudorabies virus-labeled or c-fos expressing neurons were detected in the brainstem, suggesting the enhanced supraspinal control of sphincter activity. Thus, locomotor training promotes tissue sparing and axon innervation of spinal motoneurons to improve voiding function following contusive spinal-cord injury.

## 1. Introduction

Lower-urinary-tract (LUT) dysfunction resulting from a spinal-cord injury (SCI) leads to voiding difficulty or leakage. It often causes recurrent urinary-tract infections, hydronephrosis, and ultimately renal failure, which is one of the major contributors to morbidity and mortality among SCI patients [1]. Accordingly, the recovery of LUT function is among the highest priorities in the SCI patient population [2]. Pharmacological and catheter-based approaches are the standard management of LUT dysfunction post-SCI. However, individuals either cannot tolerate the drug treatments or do not remain on long-term catheterization due to a high degree of adverse side effects and reduced quality of life [3]. Thus, there is a critical need to explore new therapeutic approaches for restoration of LUT function.

As a rehabilitation strategy, locomotor training (LT) improves urinary function after SCI in both humans and nonprimate animal models [4,5]. Several mechanisms underlie this therapeutic treatment. Among them, viscerosomatic interactions induce adaptive changes in systems other than motor within the lumbosacral circuitry, such as those controlling urogenital functions after SCI. Particularly, neurocircuits that are activated during locomotion, including the flexor and extensor reflex loop and periphery sensory input, can interact with the lumbosacral micturition reflex circuitry [5,6]. This may be mediated by spinal-cord interneuronal networks that are emphasized after SCI [7]. Systemic factors are related to the improvement of bladder function during motor exercise-based rehabilitation. SCI-induced polyuria is attributed to a reduction in antidiuretic hormone release from the posterior pituitary gland [8], and exercise mitigates polyuria symptoms [9] and reverses the negative effect on kidney function caused by increased plasma antidiuretic hormone levels [10].

Effective urination is dependent on the supraspinally controlled coordination of the bladder detrusor (DET) and the external urethral sphincter (EUS) [11]. In the clinic, approximately half of SCI patients suffer from an incomplete SCI, in which a portion of the supraspinal tract is spared to some degree. Motor training may stimulate the plasticity of this remaining spinal-cord tissue, and its interaction with lumbosacral neural circuitry improves urinary function [12,13,14,15]. Previous studies were mainly focused on LT effects on the change in bladder function [5,16], while the influence on EUS activity is understudied. Details of EUS activity displayed by electromyograph (EMG) such as the latency, frequency, and duration of the EUS bursting signal may elucidate the pathophysiology and response to LT. In the present study, we conducted LT in rats with a contusive SCI and evaluated the recovery of micturition reflexes using bladder cystometrograms (CMG) and EUS EMG recordings. Furthermore, the plasticity of the supraspinal connection to EUS motoneurons was examined.

## 2. Materials and Methods

### 2.1. Animals

A total of 36 adult male Sprague Dawley rats (weight 180–200 g, Charles River, Wilmington, MA, USA) were randomly assigned into 3 groups, namely, 12 sham, 14 SCI without LT treatment, and 14 SCI with LT treatment. Groups were scored by BBB test at 3 days after injury and then once a week until 13 weeks post-SCI. All rats received bladder CMG and EUS EMG. In each group, a pseudorabies virus (PRV-614) was injected into the EUS of 6 rats. All rats received single-bladder CMG for voiding efficiency assessment, and continuous bladder CMG and EUS EMG to examine the micturition reflex (at least 3 cycles). Animal experiments followed the NIH animal guidelines, and were approved by the Institutional Animal Care and Use Committee of Indiana University School of Medicine. Exclusion criteria: to reduce variation, animals with a BBB score of more than 2 standard deviations from the mean at 3-day post-SCI were excluded from the study [17]. After the final recording, the insertion of the electrodes into the EUS was examined, and those with inappropriate placement were excluded from data analysis, namely, 1 sham, 2 SCI only, and 2 SCI with LT treatment.

### 2.2. Contusive SCI

Under anesthetization with 2% isoflurane, the skin on the rat back was shaved and disinfected with xenodine. A drop of eye lubricant was applied to the eyes for protection. The rat was put onto the platform of an Infinite Horizon impactor (Precision Systems & Instrumentation, Lexington, KY, USA). The T10 lamina was removed, and the T12 spinal-cord segment was exposed. IH impactor parameters were set to 160 kdyn, and the impact was executed to produce a contusive injury. Then, paravertebral muscles, superficial fascia, and skin were sutured. After SCI, the bladder was mannually expressed 3 times daily until spontaneuous micturition was achieved within 2 weeks after SCI. After the first two weeks, the manual expression of urine was peformed once daily until the end of the experiment.

### 2.3. Basso, Beattie and Bresnahan (BBB) Locomotion Scale

Rats were allowed to move freely in an open circular field with a smooth, nonslip floor and were scored during a 4 min time period for their locomotion ability. Judgement was performed by two experienced members blinded to the groups. Groups were scored by BBB at 3 days after injury and then once a week until 13 weeks post SCI.

### 2.4. Forced Running Wheel Locomotor Exercise

Exercise training was conducted using a running wheel beginning 2 weeks after injury (rehabilitation conducted too early after SCI exacerbates secondary injury cascades) [18]. The exercise protocol is 60 min per session, once a day, for 5 days per week for 11 weeks. Running wheel speed started from 1 m/min and slowly increased to 6 m/min. This regimen was chosen according to the previous report that the improvement of nonlocomotory systems required longer hindlimb step training than locomotor improvements do [4].

### 2.5. Bladder CMG and EUS EMG in Fully Awake Rats

**Figure 1:**
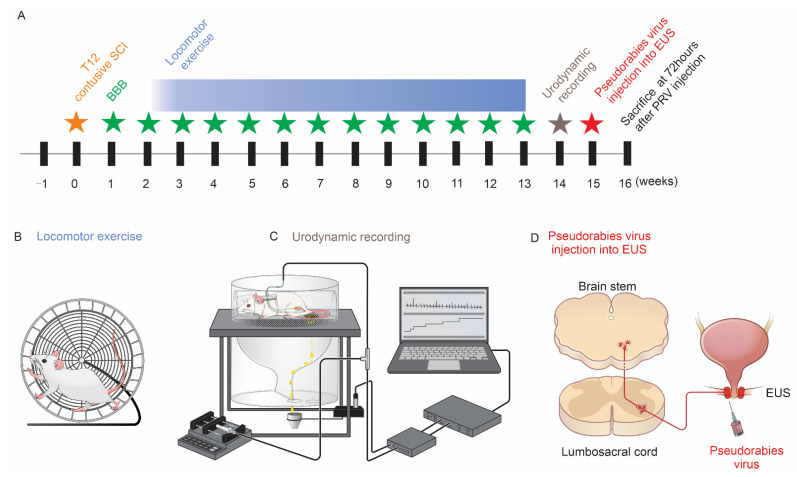
Study design diagram. (**A**) Experimental timeline and (**B**) cartoon figure of running wheel for rat exercise training after SCI. (**C**) Urodynamic recording (CMG and EMG) in an awake rat. The rat was kept in a customized restrainer. Sterile saline was instilled (0.1 mL/min) into the catheter connected with the bladder, and EUS EMG was recorded simultaneously. Voided saline was collected through the metabolic cage. (**D**) Pseudorabies virus injected into the EUS and trans-synaptically transported (retrograde) to the brainstem.

Rats were anesthetized with isoflurane. The lower abdomen was prepared, and the bladder was surgically exposed. A purse suture was created on the outer layer of the bladder wall. A sterilized polyethylene-50 catheter was directly inserted into the bladder. Sutures around the catheter were tightened. The EUS was exposed, and tungsten electrodes were embedded directly into the EUS. Electrode leads along with the catheter were subcutaneously tunneled to the back of the neck, sutured, and fixed into the soft tissue. For urodynamic measurements, the catheter was connected to a pump with a pressure transducer. Electrodes were connected to a preamplifier (HZP; Grass Technologies, West Warwick, RI, USA.) which was connected to an amplifier. The skin was then sutured. The animal was allowed to recover from anesthesia and, once fully awake and alert, was moved to a customized restrainer. A container was placed right under the animal for collecting the urine (Figure 1). We waited for approximately 60 min until the bladder function had recovered from the surgery [19]. Sterile saline was instilled (0.1 mL/min) into the bladder via the catheter [19], and all CMG and EMG parameters were recorded simultaneously. For each rat, we first performed a single CMG/EMG to examine voiding efficiency: when a void occurred, the saline infusion was immediately stopped. The volume of collected urine was measured as the voided volume, and residual volume in the bladder was measured by withdrawing through the intravesical catheter with a syringe. Bladder capacity was calculated by the sum of voided volume and residual volume. Voiding efficiency was calculated by the formula: voiding efficiency = [voided volume/(voided volume + residual volume)] × 100%. The rat was then allowed to recover again for 1 h. Subsequently, saline was continuously infused for continuous recordings of CMG and EMG to evaluate bladder and sphincter reflexes [19]. At least 3 voiding cycles were recorded in each rat. Analyzed CMG variables were peak intravesical bladder pressure (IVP), voiding threshold of IVP, micturition duration, and the number of bladder nonvoiding contractions (NVCs). NVCs were defined as small-amplitude bladder contractions without voiding during the filling phase that were greater than 8 cmH_2_O [19]. EUS EMG activity was recorded with the high-pass filter set at 100 Hz. CMG and EMG were recorded with software (Dash 8HF, V1.7.0, Astro-Med, West Warwick, RI, USA.) at a sampling rate of 2 kHz. Afterwards, recorded EMG signals were filtered in the range of 100–200 Hz using a customized script written in Matlab (R2021a, MathWorks, Boston, MA, USA.). For the EUS EMG, the latency (time between the beginning of the sharp elevation of the IVP trace and the beginning of EUS bursting activity), the bursting period during voiding, and the bursting spike number were measured. After recording, 6 of the rats in each group were anesthetized and immediately sacrificed for histological study. The 6 other rats were injected with PRV-614 into the EUS for neural tract tracing.

### 2.6. PRV-614 Injection

Supraspinal serotonergic pathways mainly originate from the caudal raphe nuclei within the brainstem. A subset of animals (6 rats/group) were used for pseudorabies virus (PRV) injection into the EUS [20]. After CMG/EMG measurement, animals were anesthetized again with 2% isoflurane. The EUS was exposed and injected with PRV-614 encoding red fluorescent protein (RFP) (Bartha strain, 10^9^ pfu/mL, courtesy of Dr. Michael A. Lane) at bilateral sites, 2 μL per site. Catheter and electrodes were pulled out from the bladder and EUS, and the bladder wall was sutured closed to avoid the leakage of urine. Muscle and skin were then closed. Animals were administered cefazolin (10 mg/kg) and buprenorphine-SR-LAB sustained release, and kept for 72 h prior to sacrifice.

### 2.7. Tissue Processing and Histology

Animals that received PRV-614 injections were kept for an additional 72 h, while others were sacrificed immediately after bladder CMG. Then, 35 μm cryostat transverse sections of brain and spinal cord were obtained. Free-floating brain sections were arranged as six serials in order.

For immunostaining, serial transverse sections were incubated in blocking buffer solution containing primary antibodies overnight at 4 °C, and then incubated in the corresponding secondary antibodies for 2 h at room temperature. Primary antibodies were against 5-hydroxytyptamine (5-HT, rabbit, 1:1000, Millipore, Burlington, MA, USA), c-fos (mouse, 1:200, ab208942, abcam, Cambridge, MA, USA), and synaptophysin (mouse, 1:1000, Millipore, Burlington, MA, USA). Primary antibody omission control was used as negative control. Immunostained sections were observed and imaged under a fluorescent microscope (Leica DM5500B, Wetzlar, Germany). Cell counting was performed under a 20× objective in one serial transverse section.

### 2.8. Masson Trichrome Staining and Quantification for Bladder Pathology

Bladder sections were incubated in preheated Bouin’s fluid and then rinsed in water. Slices were sequentially incubated in Weigert’s iron hematoxylin, Biebrich scarlet/acid fuchsin solution, phosphomolybdic/phosphotungstic acid solution, aniline blue solution, and acetic acid solution (ab150686 abcam, Cambridge, MA, USA), and then rinsed in water. Slices went through dehydration and clearing, and then were mounted. After Masson trichrome staining, collagen and muscle were colored by blue and red, respectively. Acquired photographs were separated into different single-color channels of red and blue using ImageJ software. Using the threshold function in ImageJ, the area presented in blue defined as the collagen area was measured. The area presented in red was defined as the muscle area. Collagen/muscle ratio = gray value of collagen area/gray value of muscle area. For bladder wall thickness, images were equally divided into 4 quadrants, and the thickness of the bladder wall in each quadrant was measured using ImageJ software. Bladder wall thickness is presented as the average thickness of the 4 quadrants [21].

### 2.9. Quantitative Analysis

Spared spinal-cord tissue: Sections were stained with cresyl violet (C6158, MilliporeSigma, Burlington, MA, USA). Images of the stained spinal-cord sections were taken by an Olympus BX60 microscope equipped with a Neurolucida system (MicroBrightField, Colchester, VT, USA). Morphometrical analysis of the total area of the spinal cord and the area of the spinal-cord cavity was conducted using ImageJ software. Briefly, spinal-cord sections spanning 3 mm rostrally and caudally to the lesion center were selected at 0.5 mm intervals for the measurement. The level with the smallest spared tissue area was defined as the lesion epicenter of the spinal-cord injury. The spinal cord was delineated by identifying the outer border of the spinal-cord sectioning. Then, the amount of white matter spared tissue and the lesion cavity was delineated. The area of the spinal-cord cavity was subtracted from the total area of the spinal cord. The area that was left was defined as the spared tissue area [22].

Spinal-cord neurons: On the basis of the epicenter of the SCI determined by the smallest area of spared tissue, one serial section spanning 2–3 mm caudal to the lesion epicenter was selected for quantification. Neurons in the ventral horn (VH) (most of which may be motoneurons) and neurons in the intermedial gray matter (IMGM) (most of which may be interneurons) [23,24] were counted with ImageJ software.

5-HT^+^ axons: We first identified EUS motoneurons. Onuf’s nucleus is located in rat spinal segments L5 and L6, including two separate motoneurons: the dorsolateral nucleus and the dorsomedial nucleus. In males, the dorsolateral nucleus innervates the external urethral sphincter [25]. For rats receiving PRV injection to the EUS, we identified the dorsolateral nucleus by RFP labelling. On the basis of the location from the tracing results, EUS motoneurons in the rats without PRV injection were identified with an anti-ChAT antibody. Immunofluorescence staining for 5HT was used to detect serotonergic axon terminals. Images were taken to cover the whole area of the EUS motoneurons. Images were separated into single-color channels using ImageJ software. We chose one channel according to the color of the secondary antibody used to detect 5HT for the quantification. Using the analysis tool in Image J, the average gray value of the image was measured.

RFP and c-fos positive brainstem neurons: Six serial free-floating 35 μm sections were kept in order in the cryostat protective solution. Thus, tissue sections separated by 210 μm were included in one serial of samples. Immunostained sections were imaged under a fluorescent microscope (Leica DM5500B, Buffalo Grove, IL, USA). Cell counting was performed under a 20× objective. Serotonin is crucial in mediating EUS activity [26]. According to the rat brain atlas, we chose the brainstem section between −9.16 and −14.30 mm (Bregma), which contains the raphe magnus nucleus, the raphe obscurus, and the pallidus nucleus. Cells of either RFP^+^ or c-fos^+^ colabeled with 5-HT were counted in each section and summarized in one whole serial section per rat.

### 2.10. Statistical Analysis

Statistical analyses were performed in GraphPad Prism 9. Data are presented as mean ± standard deviation of the mean. For all measured outcomes, we examined the appropriateness of the normal distribution assumption using the Shapiro–Wilk test. Normally distributed single time-point measurements between multiple groups were analyzed by 1-way analysis of variance (ANOVA). Normally distributed multiple time-point measurements between multiple groups such as BBB scores were analyzed by two-way ANOVA. When a significant overall difference was obtained on the outcome measure, Bonferroni’s adjustment was used for post hoc comparisons among the groups. Correlation between voiding function, 5-HT axon density, RFP and c-fos labeled neurons were determined. For outcome measures not normally distributed such as nonvoiding contractions, Kruskal–Wallis test followed by Dunnett’s adjustment was used in the analyses. A *p* value < 0.05 was considered to be statistically significant.

## 3. Results

### 3.1. Locomotor Exercise Promotes the Recovery of Hindlimb Function

**Figure 2:**
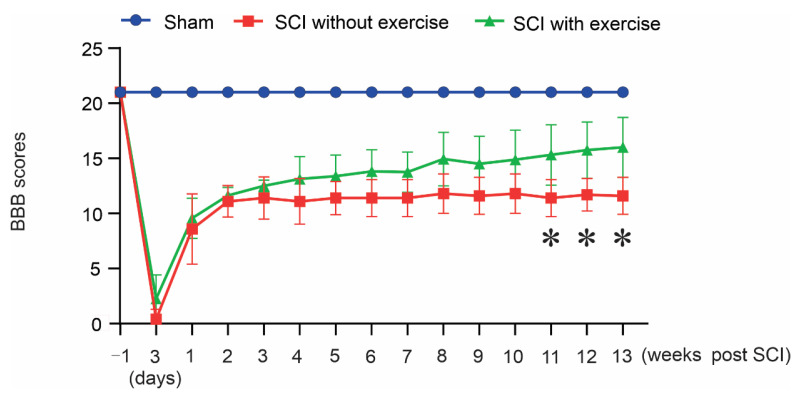
Locomotor exercise promotes recovery of hindlimb function. Improved BBB locomotor recovery found at weeks 11, 12, and 13 in the group that received locomotor exercise (*: *p* < 0.05).

We examined whether locomotor exercise led to the recovery of hindlimb locomotor function through BBB evaluation. Compared with the SCI without exercise, locomotor exercise promoted locomotor function (Figure 2). These animals showed significant differences in the score from controls in gross motor behavior at 11 weeks (sham, 21; SCI without exercise, 11.4 ± 1.67; SCI with exercise, 15.31 ± 2.74; *p* < 0.05), 12 weeks (sham, 21; SCI without exercise, 11.7 ± 1.48; SCI with exercise, 15.75 ± 2.55; *p* < 0.05), and 13 weeks (sham, 21; SCI without exercise, 11.6 ± 1.67; SCI with exercise, 16.00 ± 2.71; *p* < 0.05).

### 3.2. Locomotor Exercise Improves Voiding Efficiency

**Figure 3:**
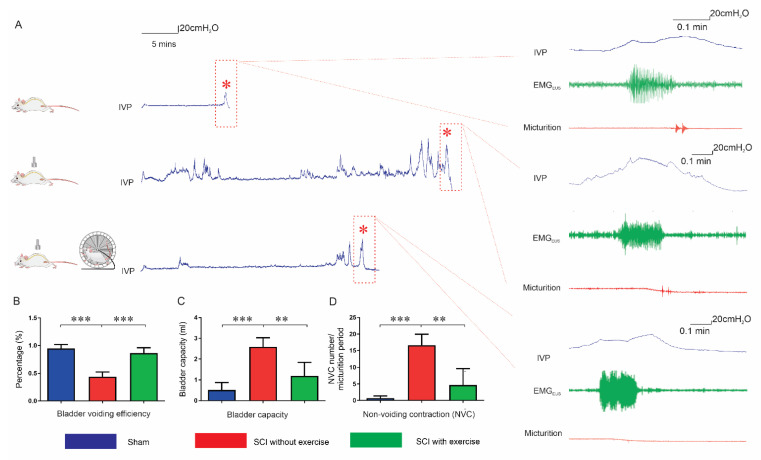
Locomotor exercise improves bladder overactivity and voiding efficiency. (**A**) Representative tracing of raw recordings in a single-bladder CMG assessment. Red asterisks, peak intravesical pressure (IVP) associated with micturition. High magnification of IVP trace within red dashed rectangle shown in right panel: (top to bottom) sham, SCI without exercise, and SCI with exercise. Each IVP trace (blue) was accompanied by a simultaneous recording of EMG_EUS_ (green), and the event of micturition (red) recorded by metabolic cage. Results indicate that, compared with SCI without exercise, exercise significantly reduces latency to induce voiding (**A**). Bladder voiding efficiency, bladder capacity, and nonvoiding contraction (NVC) were improved in the exercise group, shown in the bar graphs of (**B**–**D**). **: *p* < 0.01; ***: *p* < 0.001.

Single-bladder CMG assessment was conducted in conscious rats (Figure 3A). Voiding efficiency in SCI rats without exercise (0.44 ± 0.30) was lower than that in the sham group (0.95 ± 0.07, *p* < 0.001), and exercise enhanced the value (0.86 ± 0.10, *p* < 0.001). The bladder capacity in SCI rats without exercise (2.59 ± 1.52 mL) was larger than that in the sham group (0.51 ± 0.36 mL, *p* < 0.001). Following exercise, the parameter was significantly augmented (1.19 ± 0.65 mL, *p* < 0.001). After SCI, nonvoiding contractions (NVCs) occurred (16.61 ± 11.6 versus sham 0.7 ± 0.67, *p* < 0.001), indicating detrusor overactivity. Exercise reduced the number of NVCs (4.66 ± 4.96, *p* < 0.01 versus SCI without exercise). (Figure 3B–D).

### 3.3. Locomotor Exercise Improves Bladder and EUS Reflexes

**Figure 4:**
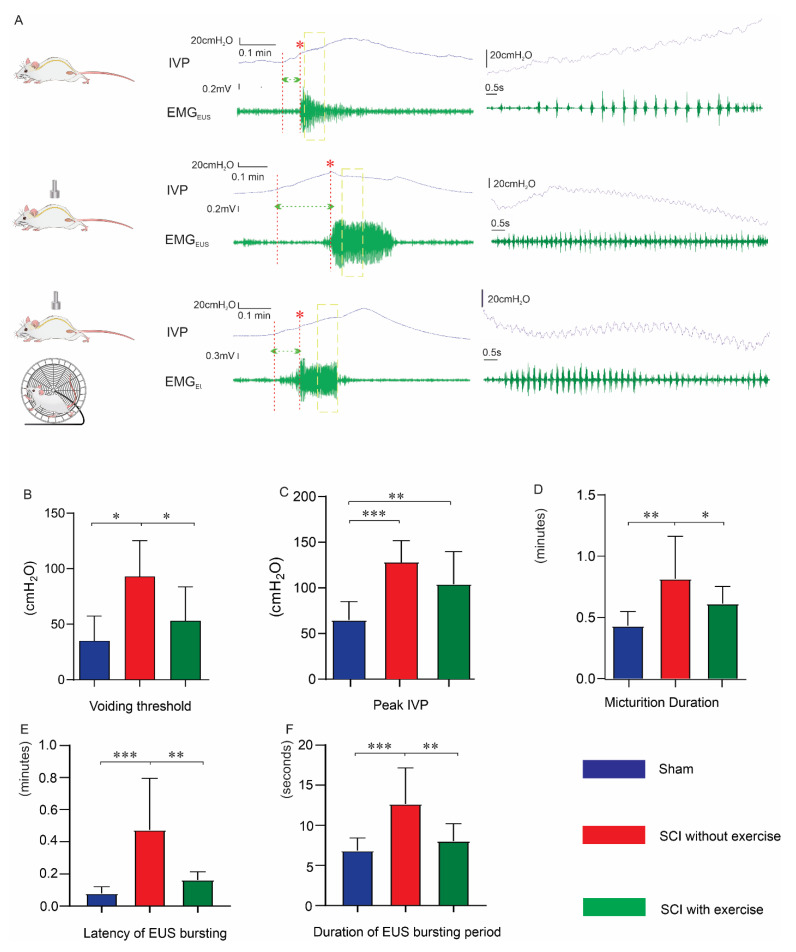
Locomotor exercise improves micturition reflex in continuous voiding recording. (**A**) In terminal awake cystometry, the representative tracing of a raw recording of continuous voiding from a rat in different groups. Each IVP trace (blue) was accompanied with simultaneous recording of EMG_EUS_ (green). Red asterisks, voiding. Micturition duration measured by time from initial pressure increase to voiding end. Green dashed lines (between two red dashed lines), latency of EUS bursting. Bar graphs, compared with SCI without exercise, exercise reduced voiding threshold (**B**), peak IVP (**C**), micturition duration (**D**), latency (**E**), and duration of EUS bursting (**F**). Time-stretched recordings within yellow boxes in each trace shown in the right panels. Each trace contains high-frequency oscillation of bladder contractions and bursting EUS activity during voiding. *: *p* < 0.05; **: *p* < 0.01; ***: *p* < 0.001.

The coordination of EUS and DET is reflected by the synchronization of bladder pressure oscillations and EUS bursting activity (Figure 4A) [27,28]. Voiding threshold is the intravesical pressure needed to initiate a void. In continuous bladder CMG and EUS EMG recordings (Figure 4A), the voiding threshold in SCI rats without exercise (93 ± 32.3 cmH_2_O) was higher than that in sham (32.2 ± 22.4 cmH_2_O, *p* < 0.05) and that in SCI rats with exercise (52.2 ± 31.1 cmH_2_O, *p* < 0.05) (Figure 4B). The peak intravesical pressure in SCI rats without exercise (129.2 ± 24.9 cmH_2_O) was higher than that in the sham group (67.12 ± 21.1 cmH_2_O, *p* < 0.001). Although exercise (104.4 ± 37.3 cmH_2_O) did not recover the peak pressure back to normal levels, there is a trend that the exercise group had lower peak pressure overall (Figure 4C).

Micturition duration in the group of SCI with exercise (0.62 ± 0.34 min) was significantly shorter than that of the SCI rats without exercise (0.82 ± 0.34 min, *p* < 0.05), and was only slightly longer than that of the sham group (0.44 ± 0.12 min) (Figure 4D). The latency of the EUS bursting signal reflects the time for the EUS to respond to the elevated IVP. The sham group had a ta latency of 0.08 ± 0.04 min. Latency in the SCI with exercise group (0.16 ± 0.05 min) was shorter than that of the SCI without exercise (0.47 ± 0.32 min, *p* < 0.01) (Figure 4E). Additionally, the duration of EUS bursting in SCI rats with exercise (8.0 ± 2.2 s) was shorter than that of the SCI without exercise (12.7 ± 4.5 s, *p* < 0.01) and not significantly different from that in the sham group (6.8 ± 1.6 s) (Figure 4F).

### 3.4. Locomotor Exercise Improves the Pathological Changes in Bladder Morphology

**Figure 5:**
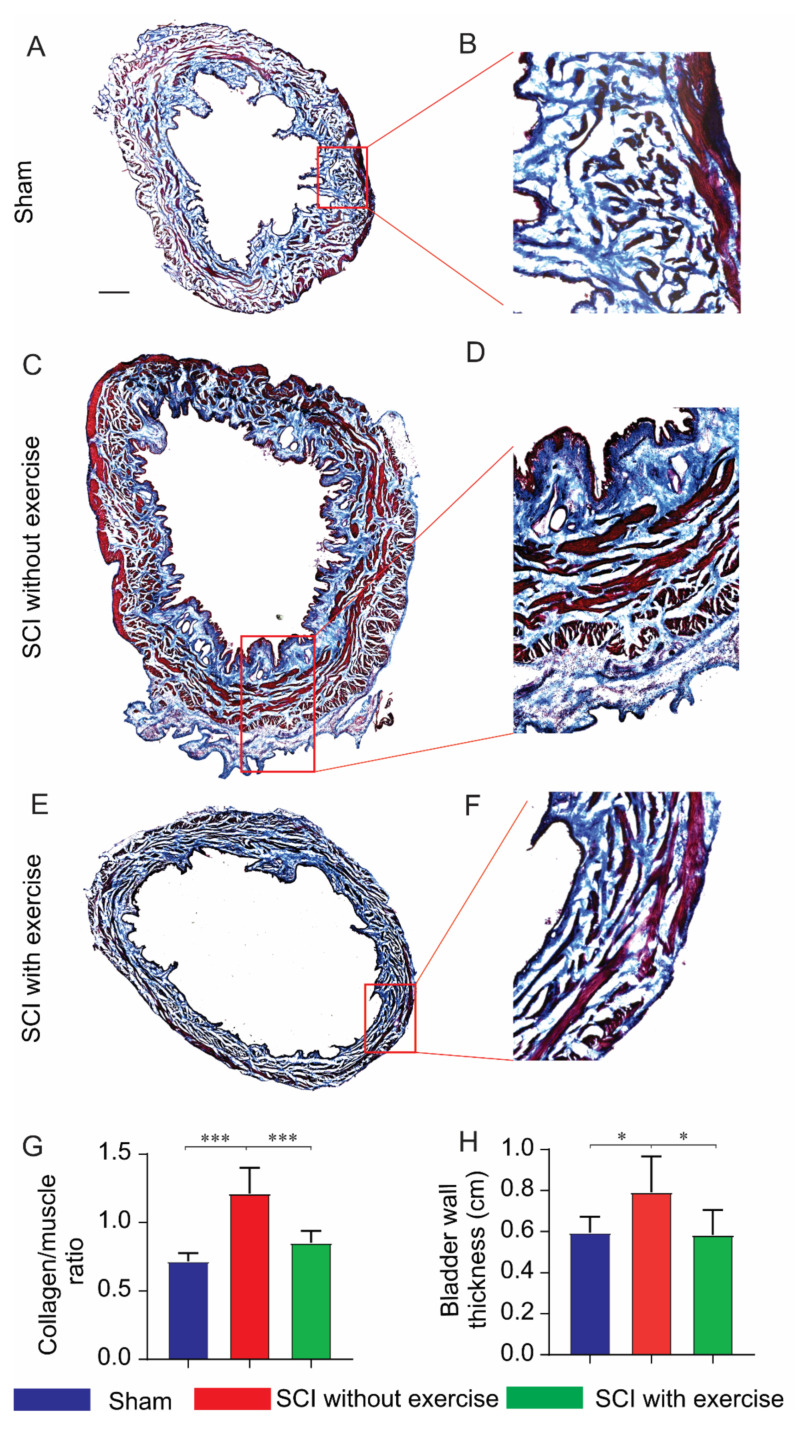
Locomotor exercise ameliorates bladder morphology. (**A**,**C**,**E**) Cross-sections of bladder stained with Masson’s trichrome in low magnification. (**B**,**D**,**F**) Areas within red dashed rectangles in high magnification. Red, muscle fibers; blue, collagen. (**G**) SCI increased the collagen/muscle ratio while exercise reduced this ratio. (**H**) SCI induced hypertrophy of bladder wall, but exercise reversed hypertrophy. *: *p* < 0.05; ***: *p* < 0.001. Scale bar 500 µm.

Morphological alterations in the bladder were evaluated using Masson’s trichrome staining (Figure 5A–F). Compared to the group of SCI without exercise, SCI rats with exercise displayed significantly improved bladder pathology. The collagen/muscle ratio was lower in the sham (0.72 ± 0.06) and SCI with exercise groups (0.85 ± 0.09) compared to SCI without exercise (1.21 ± 0.19, *p* < 0.001). In addition, the bladder wall was thinner in the sham (0.59 ± 0.08 mm) and SCI with exercise groups (0.58 ± 0.12 mm) compared to that in SCI without exercise (0.79 ± 0.17 mm, *p* < 0.05) (Figure 5H).

### 3.5. Locomotor Exercise Increases the Extent of Spared Spinal-Cord Tissue

**Figure 6:**
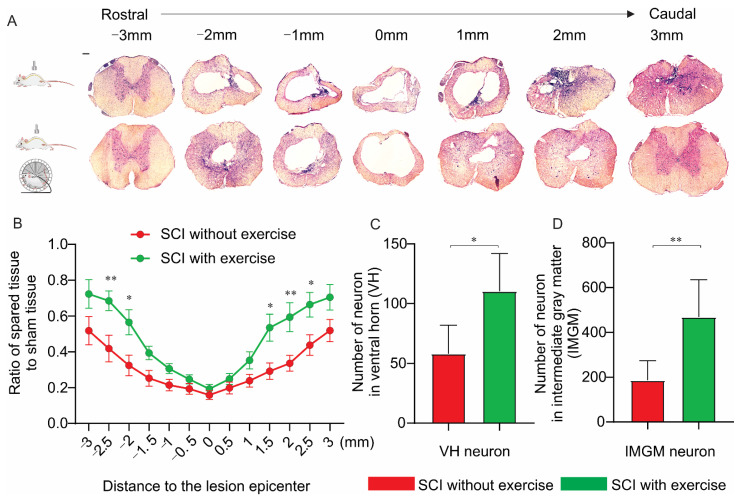
Locomotor exercise spares spinal-cord tissue. Morphometric analysis of spinal-cord lesion. (**A**) HE staining of cross-sections of injured spinal cord at various distances from injury epicenter (both rostrally and caudally) for all experimental groups at 16th week postinjury. (**B**) Percentage of spared tissue calculated by normalizing area of spared tissue to total cross-sectional area of sham spinal cord. In the lesion epicenter, there was no difference in the spared tissue between groups with and without exercise. In the location rostral and caudal to the lesion center, exercise group had more spared spinal tissue. (**C**,**D**) Group with exercise had more neurons in the ventral horn (VH) and in intermediate gray matter (IMGM) than the group without exercise did. *: *p* < 0.05; **: *p* < 0.01; Scale bar 100 µm.

To assess whether locomotor exercise promotes neuroprotection following injury, histological analysis was utilized. In the lesion epicenter, there was no difference between the spared tissue of the exercise and no-exercise groups. At 2.5 mm (*p* < 0.01) and 2 mm (*p* < 0.05) rostral to the lesion center, and 1.5 mm (*p* < 0.05), 2.0 mm (*p* < 0.01), and 2.5 mm (*p* < 0.05) caudal to the lesion center, the exercise group had more spared spinal tissue (Figure 6A,B). Consistent with an increase in spared tissue, animals in the exercise group exhibited an increase in the number of ventral horn neurons (SCI without exercise: 58 ± 24 versus SCI with exercise: 110 ± 32; *p* < 0.05; Figure 6C) and intermediate gray matter neurons (SCI without exercise: 186 ± 88 versus SCI with exercise: 470 ± 165; *p* < 0.01; Figure 6D).

### 3.6. Locomotor Exercise Promotes Serotonergic Axon Density in EUS Motoneuron Vicinity

**Figure 7:**
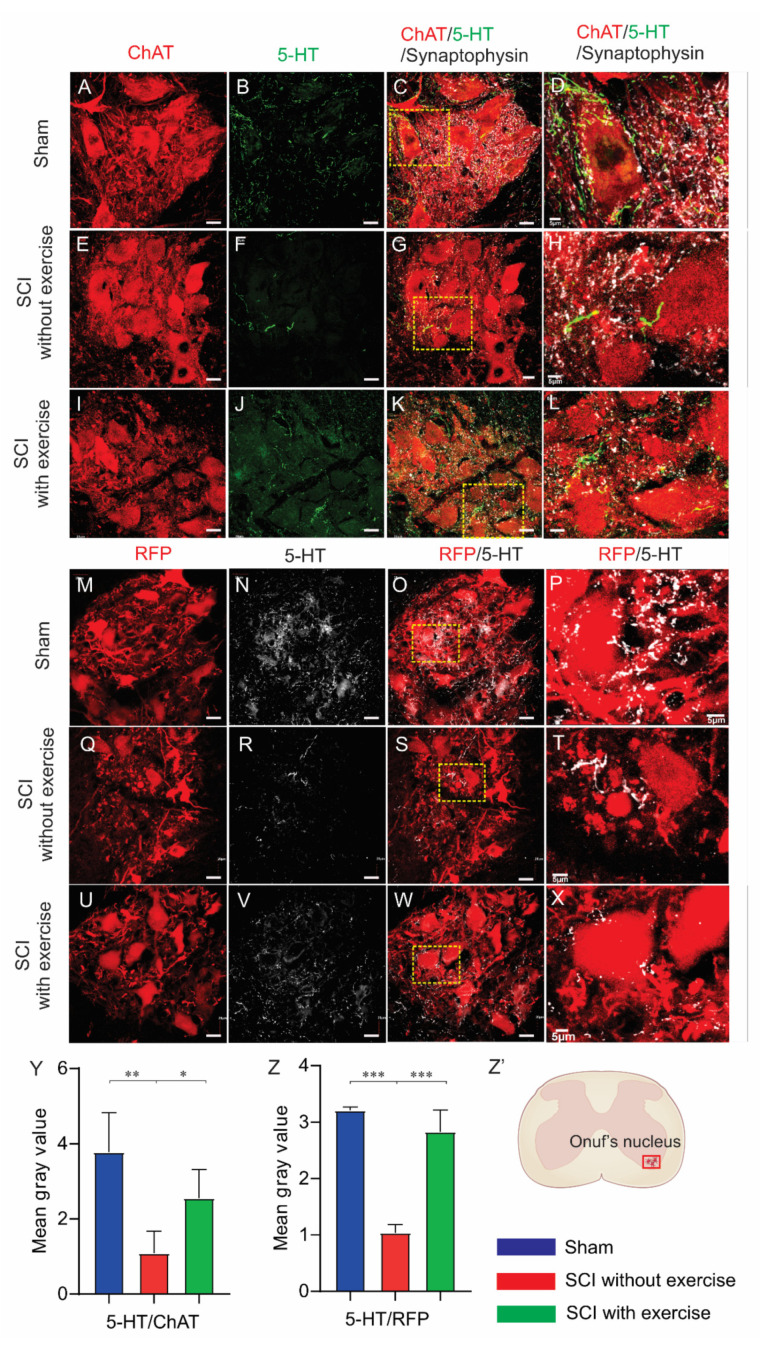
Locomotor exercise increases serotonin innervation of EUS motoneurons. Subgroup of rats injected with pseudorabies virus expressing red fluorescence protein (RFP) into EUS. Onuf’s nucleus EUS motoneurons labeled by RFP. For rats without pseudorabies virus injection, EUS motoneurons were labeled by anti-ChAT staining. Photomicrographs covering whole Onuf’s nucleus from sections in a lumbosacral cord segment costained with ChAT (red in (**A**,**E**,**I**)), (RFP, red in (**M**,**Q**,**U**)), 5-HT (green in (**B**,**F**,**J**) and white in (**N**,**R**,**V**)), and synaptophysin (white in (**C**,**G**,**K**)). Serotonin axons form synapses on motoneurons. High magnification of areas within yellow dashed rectangles in (**C**,**G**,**K**,**O**,**S**,**W**) are shown in (**D**,**H**,**L**,**P**,**T**,**X**) respectively. Result of negative control from primary antibody omission is presented as Supplementary Figure S1. (**Y**,**Z**): Compared with sham, SCI significantly reduced mean gray value of 5-HT staining while exercise training enhanced the 5-HT staining. *: *p* < 0.05; **: *p* < 0.01; ***: *p* < 0.001. (**Z’**) Area of Onuf’s nucleus EUS motoneurons where quantification of 5-HT staining was performed. Scale bars in (**D**,**H**,**L**,**P**,**T**,**X**) were 5 μm; all other scale bars are 20 μm.

To explore the effect of locomotor exercise on axonal sprouting, we colabeled supraspinal serotonin axons with antibodies for 5-HT and EUS motoneurons with either ChAT or PRV-614 immunostaining. In lumbosacral spinal-cord sections, the mean gray intensity of serotonin axons in the EUS motoneuron area in the SCI with exercise group (2.55 ± 0.77, *p* < 0.05) and sham group (3.78 ± 1.05, *p* < 0.001) was larger than that of SCI without exercise (1.09 ± 0.59) (Figure 7A–L,Y). In colabeling serotonin and RFP, the mean gray intensity of serotonin axons in the EUS motoneuron area in the SCI with exercise group (2.83 ± 0.39, *p* < 0.05) and sham group (3.21 ± 0.06, *p* < 0.001) was larger than that of SCI without exercise (1.04 ± 0.14) (Figure 7M–X,Z).

### 3.7. Locomotor Exercise Enhances Supraspinal-EUS Motoneuron Innervation

**Figure 8:**
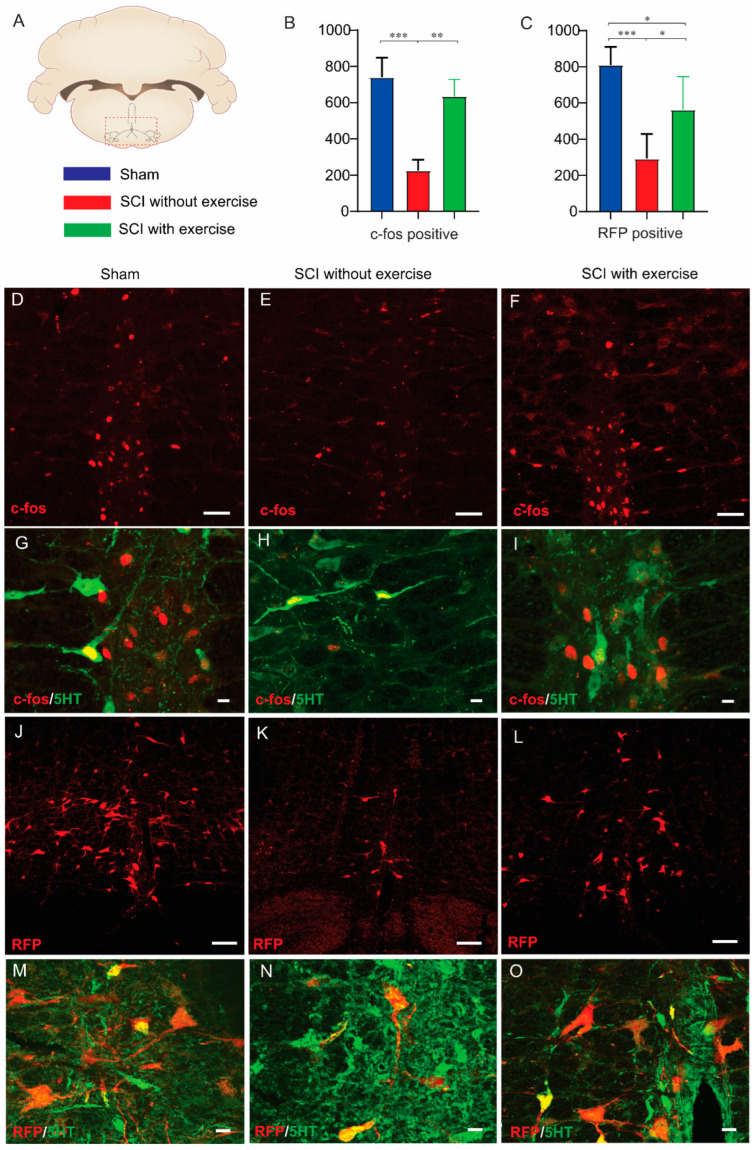
Locomotor exercise enhances supraspinal-EUS motoneuron neural circuit. (**A**) Coronal section of the brain with locations of raphe nuclei (red box area) where imaging and quantification were performed. (**B**,**C**) Number of c-fos positive cells and red fluorescence protein (RFP)-labeled neurons in SCI without exercise group were significantly less than those in the sham and exercise groups. These results indicate that exercise training recruited more supraspinal neurons to control EUS function evidenced by more activated brain stem neurons during urodynamic experiment. (**D**–**F**) Immunofluorescence staining showing c-fos positive cells in coronal section of the brain. (**G**–**I**) Some (green arrow) but not all c-fos positive neurons (red arrow) are serotoninergic (c-fos^+^, red; 5-HT^+^, green). Subgroup of rats was injected with pseudorabies virus expressing RFP into the EUS. At 72h after injection, photomicrographs from coronal section of the brain demonstrate that RFP-labeled neurons were detected in the brainstem (**J**–**L**). (**M**–**O**) Some (green arrow) but not all of the RFP-labeled neurons (red arrow) are serotoninergic (RFP^+^, red; 5-HT^+^, green). Result of negative control from primary antibody omission presented as a Supplementary Figure S1. Scale bars are 100 µm in (**D**,**E**,**F**,**J**,**K**,**L**); 20 µm in (**G**,**H**,**I**,**M**,**N**,**O**). *: *p* < 0.05; **: *p* < 0.01; ***: *p* < 0.001.

To evaluate supraspinal neurons that are involved in micturition upon induction by urodynamic recording (Figure 8A), an antibody for c-fos was used to identify neurons activated during micturition. The number of labeled neurons in the brainstem in SCI rats with exercise (635 ± 93, *p* < 0.01) and sham (740 ± 109, *p* < 0.001) was higher than that of SCI without exercise (225 ± 60). (Figure 8B,D–F). To further confirm exercise’s effect on the supraspinal-EUS motoneuron neural circuit, PRV-614 was injected into the EUS to transsynaptically infect neurons in the brainstem. The number of RFP-labeled neurons in SCI rats with exercise (563 ± 183, *p* < 0.01) and sham (810 ± 99, *p* < 0.001) was higher than that of SCI without exercise (293 ± 136) (Figure 8C,J–L). Using an anti-5-HT antibody to detect serotonergic neurons in the brainstem, results show that in rostral raphe nucleus, parts of the c-fos^+^ and RFP^+^ cells are 5-HT^−^, indicating that neurons with other types of neurotransmitters are involved in micturition (Figure 8G–I,M–O), which agrees with Ahn’s previous study [29].

### 3.8. Correlation between Supraspinal Control and Voiding Efficiency

**Figure 9:**
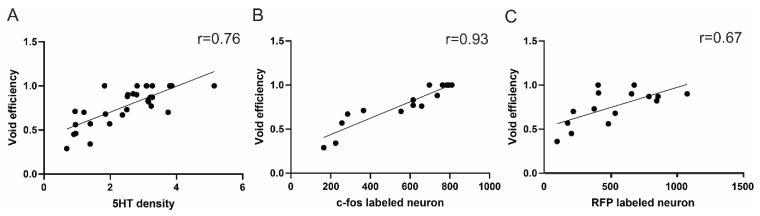
Correlation between supraspinal control and micturition function. There were good correlations between voiding efficiency and (**A**) gray value of 5-HT positive axons, (**B**) number of c-fos positive brainstem neurons, and (**C**) number of RFP-labeled brainstem neurons.

Strong correlations were shown between the supraspinal-EUS motoneuron connection and bladder voiding efficiency. The supraspinal–EUS motoneuron connection was indicated by the 5-HT positive axons in the territory of EUS motoneurons or c-fos/RFP- labeled positive brainstem neurons. r = 0.76 (5-HT/voiding efficiency); r = 0.67 (RFP-labeled brainstem neurons/voiding efficiency); r = 0.93 (c-fos/voiding efficiency) (Figure 9).

## 4. Discussion

The present study demonstrated that, following contusive SCI, exercise training preserves more spinal tissue near the lesion site, and promotes sprouting of the remaining descending axons to innervate spinal motoneurons. This causes reduced bladder overactivity and enhanced supraspinal control of EUS activity, leading to robustly increased voiding efficiency. Results uncover a novel mechanism underlying the effect of exercise on micturition in neurological disorders.

In moderate and severe contusive SCI, deprivation of supraspinal control causes the loss of voluntary micturition reflex. Although involuntary spinal reflex can be re-established over time, the emergence of bladder overactivity and detrusor sphincter dyssynergia leads to incontinence and insufficient voiding [30]. Motoneurons in Onuf’s nucleus innervate the EUS muscles. Descending projections, including serotonergic ones originating from the brainstem, are involved in the modulation of Onuf’s motoneuron activity [31]. Previous studies showed that, after SCI, the administration of a serotonergic receptor agonist improved EUS bursting period and the coordination between EUS and DET activity, leading to enhanced voiding efficiency [32,33]. In the present study, the exercise group had more dense 5-HT fibers caudal to the lesion forming synapses onto Onuf’s neurons, consistent with Engesser-Cesar’s previous report [34]

The storage phase of the urinary bladder can be switched to the voiding phase if intravesical pressure is over the voiding initiating pressure. EUS bursting signal is associated with muscle relaxation and urine discharge. With this in mind, voiding pressure and the latency to induce the bursting signal reflects how quickly the EUS responds to pressure elevation, and are thus important indicators of detrusor and sphincter coordination [35,36,37]. In our study, SCI rats had higher voiding pressure and longer latency to induce the bursting signal than the sham did. Delayed initiation of EUS relaxation caused high lower-urinary-tract resistance during urine discharge and reduced voiding efficiency. Following SCI, long-term dysuria and urine retention lead to the hypertrophy of the bladder wall with a high ratio of collagen to muscle, increasing bladder capacity. The duration of EUS bursting normally reflects voiding capability. Longer EUS bursting was unexpectedly observed in the SCI group, which might have been caused by enlarged bladder capacity, requiring more time for unsynchronized detrusor and sphincter to expel urine [37]. On the other hand, exercise significantly reduced voiding pressure and latency to initiate EUS bursting, thereby improving voiding efficiency. With bladder capacity reduced, pathological changes were improved in the bladder wall. Exercise reduced EUS bursting duration, which could be attributed to better detrusor-sphincter coordination and voiding efficiency. Nonvoiding contractions (NVCs) indicate bladder hyperactivity [38]. Here, a few NVCs were found in the sham group, which may have been due to the interference of surgical catheterization to the bladder. SCI significantly increased NVCs, while exercise reduced them. Therefore, results illustrate that exercise training ameliorates deficiencies of detrusor-sphincter coordination and improves voiding capacity.

Though it is unclear how LT promotes the plasticity of supraspinal axons, there are several possible mechanisms. Wheel running promoted a neuroprotective environment in the injured spinal cord through the induction of nerve growth factors [39,40]. Although there was no difference in the area of spared tissue in the lesion epicenter, which was similar to that in previous studies [17], the exercise group had more spared tissue in the rostral and caudal segments to the lesion epicenter. This is relevant when discussing the effects of secondary injury, which can result in neuronal damage a long distance from the original injury epicenter. LT-induced neuroprotection may not be obvious in the epicenter due to the severity of the injury. However, the increased expression of nerve growth factors induced by wheel running can be found in the lumbar spinal cord, far away from the lesion center. This implies that the neuroprotective effect of LT can be appreciated some distance from the epicenter [39]. As a result of LT-induced neuroprotection, this spared tissue provides an ideal environment for neurite outgrowth. Such outgrowth could be beneficial for rewiring circuitry with respect to micturition control. In further exploration of the potential neuroprotective effects, gene chip analysis results showed an exercise-induced increase in genes associated with myelination and dendritic remodeling [40]. Changes in genes associated with neuroprotection would likely be associated with increased fiber growth. In the present study, measurements of 5-HT intensity were an indication of both regenerative and sprouting axons. Since no 5-HT^+^ fibers were observed in the lesion epicenter, the increased number of fibers in the caudal spinal cord was likely a result of axonal sprouting but not regeneration.

In addition to the serotonergic pathway, various other neurotransmitters, such as glutamate, GABA, glycine, and catecholamine controlling micturition, were found within the intact and injured spinal cords [36]. It is reasonable to posit that the plasticity of these types of axons also contributes to micturition recovery. Despite not tracing autonomic neurons controlling the bladder, supraspinal projections likely underwent similar sprouting in the lower spinal cord. After injections of PRV into the EUS, many PRV-labeled brainstem neurons were not 5-HT+, similar to that of c-fos staining, the marker for cellular activation. This supports the assumption of sophisticated urinary control following SCI. The exercise group had more spared neurons in spinal-cord segments 3 mm caudal to the lesion epicenter. Lumbosacral spinal interneurons were identified as the basic components that modulate spinal reflex pathways [41]. Some populations of L3/4 propriospinal neurons were associated with the emergence of EUS bursting and bladder-EUS coordination after SCI [42]. Therefore, enhanced interneuron survival can provide the basis for LT-mediated LUT functional recovery.

In the neonate, lower urinary function is regulated by primitive reflex pathways organized in the spinal cord such as the perineal-to-bladder reflex during the early postnatal period. Several weeks after birth, the mature method of voiding develops, which is mediated by a spinobulbospinal bladder-to-bladder reflex pathway and by a newly emerged micturition center in the brainstem. The neonatal perineal-to-bladder reflex becomes suppressed after the establishment of these supraspinal reflexes [43]. However, the spinal micturition reflex reappears and regulates recovered involuntary urination when SCI interrupts spinobulbospinal micturition reflex pathways. The mechanism of this spinal micturition reflex is complicated, which includes the plasticity of bladder afferent pathways and the reorganization of synaptic connections in the spinal cord [44]. However, due to the limitation of our incomplete injury model that spared some supraspinal–spinal connections, the current experimental design cannot unveil the determination of the spinal micturition reflex during LT.

## 5. Conclusions

This study revealed that wheel running, a weight-bearing activity, can enhance the supraspinal–EUS motoneuron connection and improve LUT function in a clinically relevant contusive SCI model. These observations lay the foundation for future potential strategies combining pharmacological interventions, such as a serotonergic receptor agonist, with exercise to improve micturition functional recovery following SCI.

## Data Availability

Data are available from the corresponding author upon the reasonable request.

## Supplementary Materials

The following are available online at https://www.mdpi.com/article/10.3390/cells11091398/s1, Figure S1: negative control from primary antibody.
