# Supplementary figures and images for: Locomotor Exercise Enhances Supraspinal Control of Lower-Urinary-Tract Activity to Improve Micturition Function after Contusive Spinal-Cord Injury
Source: Cells. 2022 Apr 20;11(9):1398. doi: 10.3390/cells11091398 (PMC9104392; doi:10.3390/cells11091398)

Supplementary material:

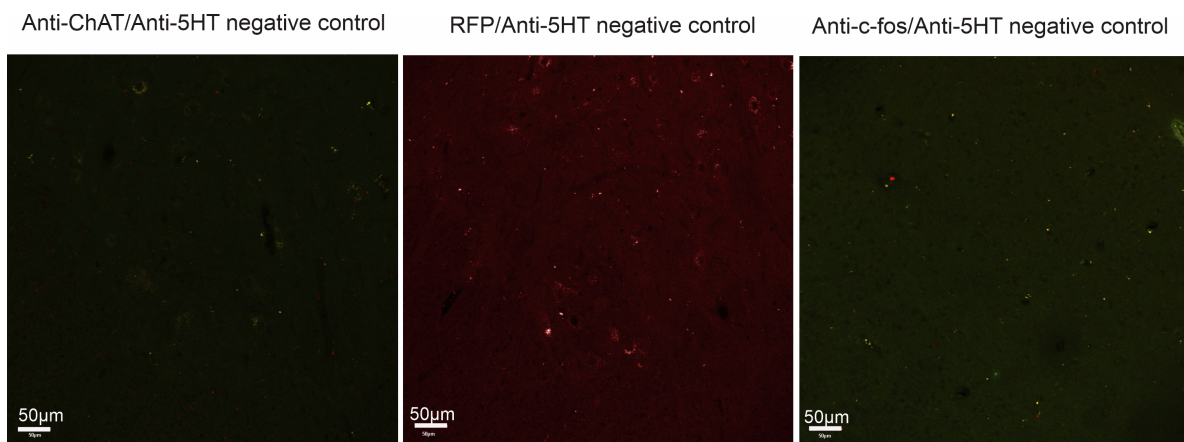

**Figure S1.** negative control from primary antibody .

Supplement: Supplementary file 1 [file cells-11-01398-s001.zip › cells-1643271-SI.pdf]
